# 阿扎胞苷联合HA方案在急性髓系白血病诱导及挽救治疗中的疗效分析

**DOI:** 10.3760/cma.j.issn.0253-2727.2022.09.011

**Published:** 2022-09

**Authors:** 降雪 侯, 凯奇 刘, 依民 胡, 述宁 魏, 广吉 张, 春林 周, 冬 林, 迎 王, 辉 魏, 尚珠 李, 建祥 王, 营昌 秘

**Affiliations:** 中国医学科学院血液病医院（中国医学科学院血液学研究所），实验血液学国家重点实验室，国家血液系统疾病临床医学研究中心，细胞生态海河实验室，天津 300020 State Key Laboratory of Experimental Hematology, National Clinical Research Center for Blood Diseases, Haihe Laboratory of Cell Ecosystem, Institute of Hematology &Blood Diseases Hospital, Chinese Academy of Medical Science & Peking Union Medical College, Tianjin 300020, China

急性髓系白血病（AML）是一种异质性很强的恶性血液系统疾病，20％～30％的初诊患者诱导化疗失败，完全缓解（CR）的患者中有相当一部分会复发[1]。这部分患者挽救治疗方案的选择需要根据一般情况、遗传学特点、前期治疗用药等确定。另外，部分初诊AML患者在确诊时身体状况较差或合并症多，只能接受低强度治疗或减低剂量的化疗方案。基于此，我们设计了阿扎胞苷（AZA）联合高三尖杉酯碱（HHT）和阿糖胞苷（Ara-C）方案（AZA+HA）用于难治复发AML患者的挽救治疗以及不适合强化疗患者的诱导治疗。

## 病例与方法

1. 病例：2016年6月1日至2020年12月30日就诊于我院的52例AML（非急性早幼粒细胞白血病）患者，初诊诱导治疗组15例、挽救治疗组37例。初诊诱导治疗组纳入经临床评估不适合应用标准剂量化疗方案诱导治疗的患者；挽救治疗组包括诱导治疗1个疗程未达CR的患者和复发难治患者。

52例患者均于初诊时行骨髓细胞形态学、流式细胞术免疫表型、细胞遗传学、融合基因和基因突变等检查。参照2017ELN标准[2]，初诊诱导治疗组中，预后良好组2例、预后中等组5例、预后不良组8例；挽救治疗组中，预后良好组1例、预后中等组29例、预后不良组7例。

2. 治疗方案：AZA+HA方案具体为：AZA 50 mg·m^−2^·d^−1^，皮下注射，第1～7天；HHT 2 mg·m^−2^·d^−1^，第4～10天，静脉输注；Ara-C 50 mg·m^−2^·d^−1^，第4～10天，静脉输注。治疗期间，依据患者临床指征给予抗感染、输注成分血、重组人粒细胞集落刺激因子等支持治疗。

CR后的巩固化疗根据患者体能状态或感染控制情况调整。包括中大剂量Ara-C方案（ID-AraC、HD-AraC方案）、标准剂量HA方案、标准剂量DA方案等。部分患者行异基因造血干细胞移植。

3. 疗效评估：早期死亡定义为诱导治疗开始30 d内任何原因造成的死亡。所有患者化疗结束21～28 d复查骨髓，结合血常规结果判断疗效。依据2017年我国成人急性髓系白血病诊疗指南[3]及2003年国际工作组的推荐[4]，进行疗效评估。复发和难治的定义参见文献[3]。

4. 随访：所有患者均随访至2021年6月30日。无病生存（DFS）时间：接受AZA+HA方案后达CR之日计算至复发、死亡、接受造血干细胞移植或随访截止日为止。总生存（OS）时间：从接受AZA+HA方案开始计算至死亡或随访截止日为止。

5. 统计学处理：采用SPSS Statistics 19.0进行数据分析。定性资料采用Fisher确切概率法进行卡方检验。定量资料以中位数（范围）来表示。

## 结果

1. 临床特征：15例初诊诱导治疗组患者中男8例，女7例，就诊时中位年龄55（14～68）岁。FAB分型：M_1_ 1例，M_4_ 4例，M_5_ 10例。37例挽救治疗的患者中，入组前1、2、3个疗程未缓解（NR）者分别为7、7、2例；复发AML 21例。男21例，女16例；中位年龄47（14～65）岁。FAB分型：M_1_ 2例，M_2_ 12例，M_4_ 5例，M_5_ 17例，M_6_ 1例。两组患者的临床特征及合并症见表1。

**表1:** 52例阿扎胞苷联合HA方案治疗急性髓系白血病患者的临床特征

临床特征	初诊诱导治疗组（15例）	挽救治疗组（37例）
性别（例）		
男	8	21
女	7	16
年龄［岁，*M*（范围）］	55（14～68）	47（14～65）
FAB分型（例）		
M_1_	1	2
M_2_	0	12
M_4_	4	5
M_5_	10	17
M_6_	0	1
WBC［×10^9^/L，*M*（范围）］	10.3（0.6～204）	10.8（1.1～129.4）
HGB［g/L，*M*（范围）］	81（32～113）	84（35～143）
PLT［×10^9^/L，*M*（范围）］	32.5（6～233）	52（4～349）
ELN危险度分层（例）		
预后良好	2	1
预后中等	5	29
预后差	8	7
核型［例（％）］		
正常核型	6（40.0）	28（75.7）
复杂/单倍体核型	1（6.7）	2（5.4）
其他核型	8（53.3）	7（18.9）
突变基因［例（％）］		
FLT3-ITD	2（13.3）	6（16.2）
NPM1	5（33.3）	13（35.1）
TP53	2（13.3）	2（5.4）
合并症（例）		
血液病史	1	1
糖尿病	3	6
高血压	3	4
感染	11	18
化疗相关脏器损伤史	0	3

2. AZA+HA方案疗效：初诊诱导治疗组3例患者出院前未评估疗效（其中2例患者化疗后因严重感染死亡）；挽救治疗组2例患者早期死亡（骨髓评价NR）。

初诊诱导治疗组总CR率53.3％（8/15），可评估疗效的12例患者CR率66.7％（8/12）。NPM1^+^组CR率80％（4/5），NPM1^−^组CR率57.1％（4/7），FLT3-ITD^−^组CR率为70.0％（7/10）。达CR的8例患者中6例（75.0％）微小残留病（MRD）≤0.01。

挽救治疗组患者均评价了AZA+HA治疗后的疗效，CR率62.2％（23/37）。1个疗程NR患者的再诱导CR率42.9％（3/7），2个疗程及以上NR患者再诱导CR率44.4％（4/9）；复发患者再诱导CR率76.2％（16/21），其中早期复发患者再诱导CR率77.8％（14/18）。初诊预后中低危组CR率为70.0％（21/30），预后高危组CR率为28.6％（2/7）。FLT3-ITD^+^组CR率为33.3％（2/6），FLT3-ITD^−^组CR率为67.7％（21/31）；NPM1^+^组CR率为76.9％（10/13），NPM1^−^组CR率为54.2％（13/24）。

3. 生存情况：初诊诱导治疗组12例患者中位随访时间为13.27（2.53～28.23）个月，1年OS率（57.1±28.6）％，1年DFS率（66.7±30.8）％，中位生存时间（17.2±13.3）个月，中位无病生存时间（16.0±12.3）个月。

挽救治疗组35例患者中位随访时间为11.67（2.23～31.53）个月，1年OS率为（70.9±16.3）％，1年DFS率（82.4±15.7）％，中位OS尚未达到，中位DFS未达到。再诱导组6例患者，其1年OS率为（80.0±35.1）％；复发难治组29例患者，其1年OS率为（70.0±17.4）％。23例患者应用AZA+HA方案后CR患者的生存明显优于NR的患者，其1年OS率明显升高［（86.1±14.7）％对（25.0±38.0）％，*P*<0.05］。

4. 骨髓抑制期：初诊诱导治疗组中，化疗相关死亡2例，化疗后所有患者均出现4级中性粒细胞减少，外周血中性粒细胞缺乏至恢复到≥0.5×10^9^/L的中位时间为11（6～21）d，PLT恢复至≥20×10^9^/L的中位时间为11（6～21）d，发热中位持续时间5.5（1～16）d；挽救治疗组中，化疗相关死亡2例，化疗后87％的患者出现4级中性粒细胞减少，外周血中性粒细胞缺乏至恢复到≥0.5×10^9^/L的中位时间为8（14～59）d，PLT恢复至≥20×10^9^/L的中位时间为11.5（3～60）d，发热中位持续时间2（0～14）d。

## 讨论

AML是一种遗传学、临床上异质性较强的恶性疾病，其化疗疗效和临床生存情况不尽相同。目前，国内年轻成人（<60岁）AML患者一线诱导化疗多为蒽环类药物联合Ara-C组成的“3+7”方案和HHT联合Ara-C为基础的方案（HAD/HAA）等[5]–[6]。应用“3+7”方案的年轻患者整体CR率53％～78％[7]。老年AML患者依据自身状况选择不同诱导方案，适宜强化治疗患者可采用标准剂量“3+7”方案。部分老年AML或少数年轻AML患者合并严重并发症，一般状况较差，无法耐受标准剂量化疗，这些患者往往采用低强度治疗方案。目前推荐的低强度治疗方案包括低剂量Ara-C、去甲基化治疗、靶向药物及以维奈克拉（Venetoclax）为基础的联合方案等[5],[8]–[10]。

复发难治AML治疗困难、预后差，对于这类患者的挽救治疗，原则上考虑由无交叉耐药的新药组成新的联合化疗方案，同时结合分子学特点（有无可及的靶向药物）、体能状态等，但目前缺乏公认、有效的再诱导治疗方案。

NCCN推荐的挽救治疗方案包括强化疗方案（如FLAG、CLAG、MEC等）、低强度治疗方案（如去甲基化药物、低剂量Ara-C、Venetoclax）以及靶向药物治疗。传统强化疗方案CR率44％～59.4％[11]–[12]；低强度治疗方案中，低剂量Ara-C CR率15％～25％，去甲基化药物（地西他滨/AZA）单药CR率20％～30％[2]，Venetoclax联合去甲基化药物/低剂量Ara-C总体CR率约为24％[13]；各种靶向药物单药/联合化疗方案疗效异质性较大，在特定的疾病类型中CR率高者可至70％，低者可至20％，且目前用药剂量及方案仍在探索中[14]–[16]。

HHT是一种生物碱类化疗药物，在国内也被广泛应用于AML的治疗[3]，以HA为基础的化疗方案在诱导化疗中取得较高的缓解率[6],[17]。在此基础上设计了AZA+HA方案，用于不适合强化疗的初诊AML诱导治疗及初始诱导NR、难治复发AML的挽救治疗。研究中可判断疗效的初诊AML患者，CR率为66.7％，且化疗后骨髓抑制期并未见明显延长；但由于患者多因并发症重采用了本方案，因此早期死亡率较高。挽救治疗的患者中，初次诱导NR的AML患者其再诱导CR率为42.9％。2个疗程以上NR患者再诱导CR率为44.4％；复发患者再诱导CR率76.2％，其中早期复发患者再诱导CR率77.8％；该方案最常见的不良反应包括4级血液学毒性及感染，绝大多数患者血常规可恢复至正常。应用该方案的挽救治疗组患者，1年OS率及DFS率均较高，其中达CR患者的预后明显优于NR患者。

综上所述，AZA+HA方案可用于无法耐受强化疗的初诊患者，也是难治复发AML患者可选择的方案。由于本研究纳入的病例数有限、随访时间较短，后续需增加队列样本量及延长随访时间进行验证。
